# Supplementary material for: Are carbon emissions trading and green financial instruments synergistic? -Comprehensive quantitative research based on content analysis
Source: PLoS One. 2024 Mar 7;19(3):e0298601. doi: 10.1371/journal.pone.0298601 (PMC10919601; doi:10.1371/journal.pone.0298601)
Supplement: S1 Data — (ZIP) [file pone.0298601.s001.zip › Data/地级市Z_F工作报告.zip!/╝¬┴╓/│ñ┤║╩╨/└┤╫╘╣1⁄2╓┌║┼í╛┬φ┐╦╩2╛▌═°í┐_2012.docx]

各位代表：

　　现在，我代表市人民政府向大会作工作报告，请予审议，并请列席会议人员提出意见。

　　一、2011年工作回顾

　　2011年是实施“十二五”规划的第一年。面对复杂多变的国内外形势，我们紧紧依靠市委的正确领导和人大、政协的监督支持，牢牢把握“三化”统筹和“三动”战略的总体要求，团结带领全市人民奋力前行、逆势进取，圆满完成市十三届人大四次会议确定的发展目标，确保实现了“十二五”顺利起步、良好开局。

　　预计全市地区生产总值完成4040亿元，增长15.2%；全口径财政收入完成800亿元，增长42%；地方级财政收入完成283亿元，增长56.5%；规模以上工业产值完成7000亿元，增长23%；新口径固定资产投资完成2500亿元，增长33%；社会消费品零售总额完成1530亿元，增长19%。这些主要经济指标的增幅在全国副省级城市中继续保持前列。

　　（一）重大项目建设取得丰硕成果，工业整体实力和水平不断提升

　　今年，我们加快实施150个重大项目，完成了1115亿元的工业投资。整车方面，丰越10万辆完成设备安装、实现试生产，大众新增12万辆产能，全市整车生产能力突破200万辆；轨道客车，长客股份二期工程投入使用，双千辆生产能力全面形成。长客装备公司搬迁改造一期竣工、二期启动建设；玉米化工，大成30万吨合成氨竣工，百万吨化工醇完成设备安装；战略性新兴产业，中航液压、国药长生、百克生物等项目开工建设，国电联合动力、兵装新能源、荷兰帝斯曼医药中间体等项目实现试生产。

　　重大项目的实施，壮大了优势产业规模，促进了产业结构调整。在国家调整汽车消费政策、汽车市场低迷不振的情况下，我市汽车工业产值仍然实现13%的增长速度；农产品加工业完成产值1020亿元，继汽车产业之后成为我市第二个产值超千亿元的支柱产业；装备制造、战略性新兴产业分别增长58.7%、32.8%，成为引领工业增长的重要力量；地方工业异军突起，对工业增长的贡献率达到61%。

　　重大项目的实施，增强了企业赢利能力，提高了工业增长质量。规模以上工业实现利润580亿元，比上年净增110亿元；完成税收360亿元，比上年净增68亿元。

　　重大项目的实施，扩大了上下游产业的市场份额，为国内外客商提供了大量的投资机会。大众发动机、丰田纺织、华信城轨等70多个投资超亿元的配套项目开工建设，富奥、纳铁福、麦格纳等20多家在长企业增资扩产，大陆电子、佛吉亚内饰、格拉默零部件、韩国KDC电子等一批研发中心落户长春。全市实际引进内资、利用外资分别增长15.5%和21.2%。

　　重大项目的实施，推动了企业自主创新，加快了科技成果转化。攻克新能源汽车制造、秸秆制糖、动车组转向架等重大关键技术130余项，500多个新产品实现工业化生产，规模以上工业新产品产值率达到45%。

　　重大项目的实施，活跃了长春经济，扩大了城市对外联系。社会物流总额达到1.2万亿元，内陆港吞吐量达到6万标箱，外贸进出口总额完成165亿美元，接待国内外游客3100万人次，这些指标均实现了15%以上的增长。

　　（二）城市化进程加快,市容环境综合整治成效明显

　　今年我们全面加快了城市建设步伐，西新区、长东北、南部新城、西部新城、铁北区域建设竞相提速，净月西部、莲花山区域开发全面启动。全市在建房产面积达到3064万平方米，相当于城区房屋存量的四分之一。至此，承载长春未来的城市框架全面拉开，主城区大建设、大发展的态势已经形成，长春大踏步地进入了城市化的高潮期。

　　围绕老城区改造提升，开展了第二轮150天市容环境综合整治行动。这次整治的力度更大、效果更明显。

　　市容整治，拆除违法建筑2.2万处、74.5万平方米，改造10条特色街路，完善提升5个传统商圈、15条精品街路，主城区主要街路楼宇基本完成整修。

　　历史街区，实施了南广场、人民大街站前段、胜利大街一期、铁南街区改造工程，南大营、沙俄领事馆等20座历史建筑按原貌完成修复。

　　绿化建设，新建大块绿地67块，彩化街路100条，新植街路75条。新建续建11个公园，9.7平方公里的长东北湿地公园等5个公园对游人开放。

　　生态建设，新一轮伊通河综合整治顺利推进。石头口门、净月潭、新立城生态湿地面积进一步扩大。节能减排指标均完成年度任务。空气质量优良级天数继续保持在340天以上。

　　环卫保洁，主要街路、重点商圈和商业街实现全天候保洁，四环路以内增设移动式垃圾转运站219个，城乡结合部主要村屯卫生清扫实现城区化管理。

　　市政道路，大中修道路164条，维修养护道路929条，3座跨伊通河大桥、109条道路竣工通车，远达大街、机场大道、北凯旋路、东自由大路、东吉林大路建设顺利推进。

　　轨道交通，西客站综合换乘中心主体封顶，长春站综合换乘中心北广场交付使用。轻轨4号线投入试运营，轨道交通形成环线。全市人民期盼多年的地铁工程开工建设。

　　交通管理，连续实施7期交通调流，开辟10条公交专用车道，安装81公里道路隔离护栏，新设142条单行线，调整限制路口200余个。在全年新增10.1万辆新车的情况下，城市交通基本保持通畅。

　　城市管理，深入整治野广告、露天烧烤、渣土清运、静态停车等城市管理顽疾，全面推行网格化管理模式，城市管理长效机制进一步完善。坚持依法管地用地，节约集约利用水平明显提高。

　　（三）农业喜获特大丰收，县域发展实现新突破

　　粮食产量突破200亿斤，创历史最高水平。启动实施了30万亩高标准粮田、98个万亩高产示范田建设工程。玉米保护性耕作示范推广面积达到30万亩。玉米螟生物防治实现全覆盖。水稻机械化栽培技术全面推广。人工增雨三期、防雹网二期工程建成投入使用。建设新菜田2.1万亩。新建200个牧业小区，连续9年无重大动物疫情。

　　完成了20万亩旱田节水灌溉工程，新建续建一批农田水利设施，高标准修复2010年汛期水毁的水利设施，完成水利投资比去年增长近一倍。

　　新增农用机械5万余台（套），农机总动力增长8.9％，农机化综合水平达到70％。机械替代人工成为农业耕作的主要手段，进一步解放了农村劳动力，农村外出务工经商人数突破百万大关。

　　县域工业加快发展，实施投资超3000万元的工业项目250个，工业投资增长23%，规模以上工业产值增长20.2%。

　　县域财政实力明显增强，农安突破20亿元,九台达到18.2亿元，德惠、榆树和双阳均超过10亿元，13个乡(镇)成为财政收入亿元乡（镇）。

　　四县（市）新城建设全面启动，房地产开发规模平均达到百万平方米，一些重点乡（镇）达到10万平方米，城镇化开始成为县域发展的新动力。

　　新农村建设扎实推进，营造林5200公顷，新增标准化储粮仓5万套，新建农村公路1026公里，绿化美化村屯330个，25万农村群众饮水安全问题得到解决，农村泥草房改造基本完成。

　　（四）现代服务业提速增效，中心城市功能不断完善

　　为突出发展现代服务业，今年我们出台了一系列新的扶持政策，一次性开工50个投资超5亿元的大项目。现代服务业完成投资674亿元。服务业增加值增长13.9%，比去年提高1.3个百分点。服务业对经济增长的贡献率达到37.3%，比去年提高2.9个百分点。

　　高端服务业项目竞相涌入南部新城核心区、长东北核心区、净月彩宇大街，落地项目总数达到37个，建筑面积765万平方米，总投资近400亿元。这三大区域的启动建设，将带动现代服务业快速成长，集中展示长春繁荣、时尚、现代的新形象。

　　新启动9个商务综合体，全市规划建设的商务综合体总数达到24个。这些商务综合体，单体规模均超过20万平方米，集餐饮、娱乐、文化、休闲、购物、办公等功能为一身。建成后，不仅繁荣商贸、方便群众，还将进一步优化城市功能布局和交通结构。

　　尚德森铭、动漫软件等一批文化产业园区开工建设，各类文化企业发展到1.7万户。文化产业实现增加值301亿元，占GDP比重提高1.6个百分点。

　　盛京、华夏2家股份制银行落户长春，引进组建风险投资等各类基金公司11家、小额贷款公司19家。

　　成功举办汽博会、农博会、民博会、雕塑展、冰雪节、消夏节、书博会、创业博览会等190余项会展活动，促进了相关产业发展，丰富了城市文化生活。

　　中机物流、工业品交易中心等物流批发大市场竣工建成。兴隆综合保税区正式获得国家批准，长春拥有了一个直接面向世界的开放平台。

　　（五）民生工作扎实推进，人民群众生活进一步改善

　　今年，我们把市级财政70%以上的新增收入用于改善民生，又为群众办了一批好事、实事。

　　新增城镇就业11.1万人，“零就业家庭”保持动态为零，城镇登记失业率为3.3%。职工最低工资标准、企业退休人员养老金、失业保险金月均提高150元以上。减免个体工商业户、小型微型企业税费4亿多元，4.9万户经营者年均增收8000元左右。城市居民人均可支配收入20400元、农民人均纯收入7400元，分别增长14%和12%。

　　通过实施政府助保，5000余名困难职工接续养老保险，1100余名老知青、1.4万余名“五七家属工”办理养老保险手续。城镇企业职工养老保险新增参保12.8万人，失业保险新增参保10.5万人。住房公积金当年归集额突破60亿元。10万名改制企业退休职工社保、医保、补贴发放等相关服务与原企业脱钩，实现社会化托管。新型农村养老保险试点在全市铺开，50万余名60岁以上农民开始按月领取养老金。

　　年内先后两次提高城区低保补助标准，城镇低保月保障标准提高到375元，农村低保年保障标准提高到2100元，分别增长23%和40%。四县（市）低保保障标准均有不同幅度的提高。低保家庭幼儿和义务教育阶段学生低保金上浮20%。启动了低保补助标准和物价水平挂钩联动机制，从4月份开始连续6个月为14万困难群众发放临时物价补贴。

　　700余名城区低保、低保边缘家庭新入学大学生分别获得10000元和5000元的助学补贴。830余名贫困家庭大学生人均获得6000元左右的生源地助学贷款。深入开展“万户特困户结对救助”活动，17000多户贫困家庭得到多种形式的帮扶。

　　全面加大贫困家庭残疾人救助力度，24名聋儿实施人工耳蜗手术，30名孤独症儿童获得康复治疗补贴，146名肢残者安装假肢，418名残疾学生和贫困残疾人子女获得扶残助学金，精神病防治、残疾人家庭无障碍用具配发基本实现“有一助一”。

　　新建5家公办养老机构、7家民办养老机构，新增养老床位3018张，全市千名老人拥有养老床位数达到29.6张。318个城市社区全部建成居家养老日间照料站。五城区农村社区公共服务中心实现全覆盖。

　　2287栋、1060万平方米老旧楼宇完成外墙保温、楼顶防水、地沟清掏、管网平衡改造，数量相当于去年的2倍，60多万群众受益，“暖房子”工程成为广受市民赞誉的民生工程。30个“老旧散”小区实施环境、绿化、道路综合改造，旧貌换新颜。

　　开工建设6433套廉租房、11439套公租房、12950套棚户区回迁房，分层次、多形式的城市住房保障体系基本形成。拆除城区D级危房320栋。无籍房确权200万平方米。集中建设了240万平方米农民回迁小区，2.8万户被征地农民喜迁新居，基本实现先回迁、后拆迁。

　　全面开展市政公用设施“冬病夏医”活动。新建5座变电站。改造150处二次供水泵站。并网改造小锅炉房302座，新增供热能力3100万平方米。改造燃气高危管网352公里，相当于过去6年的总和。城区公交车全部安装取暖设施，市民冬季出行有了暖公交。今年入冬以来，电、水、气、热等方面投诉总量下降21%，群众满意度明显提升。

　　（六）文明城创建终获佳绩，社会事业全面进步

　　今年，我们进行了全国文明城市创建工作的最后冲刺。在全市人民的共同努力下，长春在异常激烈的竞争中脱颖而出，被正式命名为“全国文明城市”。这是反映城市整体文明水平的最高奖项，是长春最具价值的无形资产。长春人为生活在这样的城市而感到骄傲和自豪。

　　各级各类教育协调发展。制定出台了学前教育三年行动计划，新建17所公办幼儿园。新建、加固中小学校舍100万平方米。提高了农村中小学、少数民族学校和特殊教育学校公用经费标准。孤独症康复训练中心建成竣工，进一步完善了特殊教育体系。汽车工业高等专科新校区投入使用，农业学校异地新建工程全面启动。完成各类职业培训50万人次，为经济社会发展输送技能型人才4万余人。

　　医疗体制改革扎实推进。大幅度增加公共卫生投入，5家区级医院、56个社区卫生服务中心、137个乡（镇）卫生院基本药物与零售药店实现“同城同价”，药品价格下降29.2%、住院费用下降21.3%、就诊患者人次上升10.7%，基本药物制度改革走在全国前列。在全国率先实施医保“议价谈判”，阑尾炎等10个病种患者个人支出平均降低53%，3233个常规诊疗处置项目费用平均降低23%。城镇居民医保、新农合报销比例分别提高5个和10个百分点。4万多对孕前夫妇接受免费优生筛查，筛查项目由6项增加到19项，高风险生育人群全部落实干预措施，从源头提高了出生人口素质。新建献血屋12座，自愿无偿献血量增长13%，确保了城市用血安全。

　　长春被文化部、财政部确定为首批创建国家公共文化服务体系示范区城市。市博物馆、规划展览馆、文化艺术展览馆、长影老厂区、市图书馆新建改造工程进展顺利，70个乡（镇）综合文化站投入使用，马孔德雕塑艺术馆、科技文化中心部分展馆建成并向游人开放。

　　全民健身活动蓬勃开展，市民身体素质进一步提高。奥林匹克公园开工建设。南湖冬泳基地建成投入使用。全国“十二冬会”长春赛区筹备工作基本就绪。

　　（七）政府依法行政水平不断提升，社会保持和谐稳定

　　自觉接受人大及其常委会法律监督、工作监督和政协民主监督，坚持重大事项向人大报告、向政协通报制度。认真听取人大代表、政协委员和各民主党派、工商联以及无党派人士的意见和建议。办理人大代表建议195件、政协提案337件。

　　开展12次局长接待日活动，42个政府部门，15个县（市）区、开发区及其下级部门，市政公用单位及其分支机构，490多个单位的主要负责人开门接访，累计接待来访群众2万余人、解决各类问题近7000个。局长接待日已成为政府面对面听取群众意见、解决民生问题的长效机制。全面加强信访工作，扎实办好市长公开电话，主动接受新闻媒体监督，全市信访总量降低20%，已经连续4年大幅度下降。

　　严厉打击各类刑事犯罪，命案破案率、交通肇事逃逸案破案率保持在90%以上。抓获公安部上网通缉逃犯1547名，数量相当于去年的2倍，市公安局荣获公安部评定的集体一等功。“天网工程”新增监控探头2.7万个，总量达到10万个。7月份开始实施公安夜巡，千余名干警对主城区实施全方位巡查。这些措施对犯罪分子形成了强大震慑，110有效警情、刑事案件立案率分别下降54%和15%，社会治安状况进一步好转。

　　扎实推进“安全建设年”活动，排查整改安全隐患5万余个。深入实施药品、豆制品、添加剂、瘦肉精、成品粮、肉类食品等专项整治行动，食品药品安全状况有所好转。依托民兵、预备役建立6支抢险队伍，消防特勤二大队完成组建，城市应急抢险救援能力进一步提升。

　　深入开展创先争优、“三满意”机关创建、“万人评议机关”等主题实践活动，政府各部门推动发展、服务群众、促进和谐的能力不断提高。

　　严格依法行政，深入推进政务公开，规范行政执法行为，压缩审批时限，规范各类收费，软环境质量进一步提高。

　　坚持从严治政，廉政建设和反腐败工作取得新成效。

　　双拥模范城创建工作通过国家考核验收。

　　国家安全、统计、民族、宗教、司法、外事、侨务、档案、保密、人防、地方志、红十字等工作都取得了新成绩。

　　各位代表，一年来所取得的成绩，凝聚着全市各族人民的心血和汗水，体现着各方面的帮助和支持。在这里，我代表市人民政府，向为长春发展做出无私奉献、付出辛勤劳动的全市各族人民致以亲切的问候和崇高的敬意！向给予我们支持与监督的人大代表、政协委员，各民主党派、工商联和无党派人士、人民团体，向参与长春发展建设的中省直单位、驻长部队指战员、武警官兵和港澳台同胞、海外侨胞及国际友人表示衷心的感谢！

　　总结一年来的工作，我们也清醒地认识到，经济社会发展和政府工作中还存在许多问题：

　　——工业发展空间不足问题初现端倪，生产要素供应滞后发展的矛盾比较突出，科技创新对发展的支撑带动作用不强，加快转变发展方式十分迫切；

　　——城乡居民收入总体水平不高，生活必需品价格上涨幅度较大，中低收入群众生活受到影响，部分群众生活还很困难，改善民生任重道远；

　　——政府贷款融资受限，城市建设资金严重短缺，部分道路建设改造项目实施进度受到影响，机动车持续高速增长，城市交通压力有增无减；

　　——安全基础设施仍然较为薄弱，事故隐患排查整改需要进一步加强，城市安全事故高发的势头还没有从根本上得到扭转；

　　——政府职能转变不够，行政效率需要进一步提高，个别工作人员公仆意识、责任意识和服务意识不强，官僚主义和腐败现象仍然存在。

　　这些矛盾和问题，需要我们认真面对，下决心予以解决。

　　二、2012年主要任务

　　明年经济社会发展的环境仍然充满困难和挑战，但长春有自己独特的优势，具备稳中求进、好中求快的基础和条件。我们的市场主要在国内，受国际经济形势的直接影响比较小；经济支撑主要是大企业，抗风险和波动的能力相对强；连续多年高强度投资，为经济社会发展积蓄了强大势能；汽车、轨道客车、农产品加工等主导产业的市场需求，仍处于上升期；特别是有省委、省政府和市委的正确领导，有全市人民昂扬向上的精神状态，我们一定能够保持又好又快的发展势头，把长春的振兴发展大业不断推向前进。

　　明年政府工作的指导思想是：深入贯彻科学发展观，以科学发展、加快发展、率先发展为主题，以转变经济发展方式为主线，紧紧围绕加快发展、改善民生、建好城市、促进和谐大局，坚定不移地推进工业化、城镇化、农业现代化“三化”统筹，坚定不移地实施投资拉动、项目带动、创新驱动“三动”战略，坚定不移地推动开发区、城区、县域“三大板块”协调发展，坚定不移地走工业和服务业双拉动增长路径，坚定不移地推动文化大发展大繁荣，坚定不移地推进富民工程和社会事业发展，坚定不移地加强精神文明和民主法制建设，加快打造全省科学发展的领头羊、民生改善的排头兵、社会和谐的首善区，努力让城乡居民生活得更加美好。

　　明年全市经济社会发展的主要预期目标是：地区生产总值增长14%左右，全口径财政收入实现同步增长，固定资产投资增长20%以上，规模以上工业产值增长18%左右，服务业增加值增长15%左右，社会消费品零售总额增长20%左右，城市居民人均可支配收入增长14%以上，农民人均纯收入增长12%以上。

　　重点抓好以下七项任务：

　　（一）加快工业转型升级

　　明年，我们要以实施150个重大项目为载体，进一步做强实体经济、调整产业结构、转变发展方式。

　　坚定不移地建设三大世界级产业基地。丰越10万辆正式投产，力争启动通用二期、奥迪扩能、轿股二厂、明君商用车4个整车项目；百万吨化工醇投产运行，第二个百万吨化工醇全面启动；长客装备公司搬迁改造二期竣工投入使用。加快实施百万台发动机、百万台变速器、百万套传动轴、百万吨聚酯以及轨道客车轮对、变速箱等一批上下游项目，向产业链要产值、要效益、要竞争力。

　　大力发展先进装备制造、光电信息、生物医药、新材料、新能源五大战略性新兴产业，推进高精度电机、LED显示屏、光纤激光器、生物疫苗、风电机组、铝镁合金等一批重点项目，加快培育100户销售收入超亿元的高技术企业。

　　依托高校科研院所科教资源优势，加快科技成果转化，建设创新型城市。新建一汽乘用车等一批研发中心。长东北科技创新中心落户国家级科研机构20家以上，百万平方米科技企业孵化器建成投入使用。

　　支持骨干企业采用先进的质量和产品标准，争创一批国家驰名商标。继续实施中小企业扶持工程，着力突破融资难等瓶颈制约，新增中小企业2000户以上，民营经济增加值增长20%以上。

　　充分发挥长吉图和长吉一体化战略的旗帜作用，吸引央企和战略投资者以更快的速度向长春集聚。实际利用内资、引进外资分别增长20%左右。

　　兴隆综合保税区封关运营，启动建设国基电子等10个项目。积极推动高新技术产品和工业制成品出口，促进出口结构升级，外贸进出口总额增长15%以上。

　　努力抓好经济运行，确保工业持续增长，稳定全市经济大局。

　　（二）扎实推动县域经济突破

　　毫不放松地抓好粮食生产，继续创建98个万亩高产示范田，抓好200万亩全程机械化耕作示范区建设，100万亩玉米实现保护性耕作，玉米螟和稻瘟病力争实现统防统治。启动建设乐山、莲花山都市观光农业示范区。全面加强气象防灾减灾工作，进一步扩大农业保险覆盖面。正常年景下粮食产量保持在180亿斤以上。

　　抓好“菜篮子”工程，当年新增棚膜菜田2万亩。增加蔬菜交易场所，扩大农超对接范围，加快建设鲜活农产品流通体系。

　　新建100个牧业小区，大力推广标准化生态养殖技术。加强重大疫病防控。基本完成乡（镇）畜牧兽医站达标改造。

　　大兴农田水利建设，实施饮马河等3处大型灌区配套改造，新建150个旱田节水灌溉作业区，完成一批病险水库除险加固。

　　大力发展农业机械化，培育新型农村合作经济组织，健全农业社会化服务体系，积极稳妥地推动土地规模经营。

　　四县（市）要以培育农副产品加工业和承接城市产业转移为重点加快壮大工业园区，工业投资、工业产值都要增长20%以上。

　　高标准改造建设4个县城，推动7个重点乡（镇）城乡双向一体化试点，大力发展各项社会事业，全面提升服务能力和承载能力，吸引农村人口向城镇集聚。

　　加快建设167个省级新农村示范村，绿化美化村屯200个，建立村村通公路养护长效机制，新建5万个标准化储粮仓，营造林3000公顷，解决15万农村群众饮水安全问题。

　　（三）大力发展现代服务业

　　明年，在整个经济工作的摆布上，我们要进一步突出现代服务业。现代服务业增速要达到20%以上，成为全市经济增长的新引擎。

　　金融保险、现代物流、商务综合体、总部经济、工业设计和服务外包等重点领域，都要明确具体的支持政策，集中实施一批重点项目，力求实现大的突破。

　　加快建设南部新城核心区、长东北核心区、净月彩宇大街三大高端服务业聚集区，在建项目数量力争达到50个以上。再启动建设一批商务综合体，并以此为依托建设城市新的商业次中心。

　　支持国内外企业在长设立销售中心、结算中心和研发中心。大力引进域外股份制银行及知名中介机构、区域性总部。建设喜来登、希尔顿、凯悦等五星级酒店。力争2到3家企业上市融资。

　　加快传统商贸服务业晋档升级，大力发展电子商务等新兴业态，提高消费对经济增长的拉动作用。积极开发旅游资源，不断壮大会展业，旅游业和会展业总收入均增长20%以上。

　　今后，五城区和净月、莲花山开发区，经济工作的主要任务就是发展服务业，都要成为服务业发展的主力军。以工业为主的开发区，既要大力发展生产性服务业，也要成为所在区域的商业中心、商务中心。

　　（四）着力建设绿色宜居城市

　　明年，我们要以更大的力度推动城市建设与改造。经开与九台、高新与德惠要合作建设新区，推动城市向东北方向拓展。南部新城、长东北、净月西部、西部新城、莲花山、西新区绕城高速以外区域都要完善基础设施，加速人口、产业和要素集聚，尽快成为现代化的新城区、产业区。

　　继续开展市容环境综合整治，完善提升精品街路、标准化街路，优化改造传统商圈，进一步提高城市精细化管理水平。

　　加快建设七大城市生态湿地，新建续建8座公园，新建63处大块绿地，新植21条街路，彩化100条街路，城区绿化面积增加500公顷。

　　主要街路保洁基本实现机械化作业，背街小巷清扫保洁达到干路标准。

　　启动建设“两横两纵”快速路，年内实施西半环建设，做好东半环开工准备。拓宽三环路，续建机场快速路、东自由大路、东吉林大路，新远达大街全线通车。实施一批重要交通节点的立体化改造。打通一批断头路，进一步畅通路网微循环系统。

　　轻轨4号线正式运营，西客站综合换乘中心投入使用。地铁1号线全线开工，力争开工建设2号线，尽快形成十字形的地下轨道交通骨架。继续更新公交车辆，增设公交专用车道，适当延长公交线路，合理布局公交站点，更好地满足市民出行需要。

　　继续实施交通调流，增设一批单行线、50公里道路隔离护栏，提高交通事故处理效率，强化动静态交通管理，完善行人过街设施，尽快实现交通管理智能化。

　　改造长白公路、北亚泰大街城市出入口，实施长深、长平、长吉等高速公路新建扩建工程，改造提升长白、长伊、长石等一批公路等级。

　　加快实施新一轮伊通河综合整治，提升改造北郊、南郊污水处理厂，新建两甲污水处理厂，东南、兴隆污水处理厂投入运行，改造4条城市明沟，整治粉煤灰厂等点源污染，城区段力争全线蓄水、水质基本达标。

　　改造扩建现有水厂，五水厂建成通水，筹建六水厂。新建热电五厂，扩建7座区域锅炉房，并网改造100座小锅炉房，新增供热能力1500万平方米以上。新建改造2座输气站、22座二次变电站。

　　完成地下管网普查，改造建设60公里供水管网、81公里供热管网、100公里排水管网、320公里燃气管网，尽快形成完善可靠的地下管网系统。

　　深入开展“联合保护城乡环境，共建绿色宜居城市”活动，全面完成节能减排任务，不断提升生态环境质量。增强依法管地用地意识，严肃查处违法用地，努力提高土地资源节约集约利用水平。

　　（五）切实加强文化建设

　　文化是城市的根基和灵魂。我们必须以高度的文化自信和文化自觉，全面加强城市文化建设，更加主动地迎接文化的大发展、大繁荣。

　　全面创建国家公共文化服务体系示范区。启动建设群众文化活动中心、朝鲜族艺术馆。支持省图书馆新馆建设，市图书馆、市少儿图书馆完成维修改造，新建20个市级图书馆分馆。14个县级图书馆、14个县级文化馆和66个街道综合文化站、97个乡（镇）综合文化站、1687个村文化活动室年内达到国家规定标准。

　　继续改造铁南历史街区，实施胜利大街二期改造，改扩建孔子文化园，长影老厂区完成改造对外开放，修缮一批有价值的历史建筑。启动建设月亮岛文化园。加快建设市博物馆、规划展览馆、文化艺术展览馆、汽车博物馆。鼓励社会力量兴建一批行业博物馆、收藏博物馆。

　　大力发展文化创意、数字出版、动漫游戏等新兴行业，壮大一批文化产业园区，扶持100户文化骨干企业，尽快把文化产业培育成新的支柱产业。

　　电影节、消夏节、汽博会、农博会等重点展会都要增加群众文化活动，都要争取办成市民的节日。办好文化艺术周、农民文化节、大学生艺术作品展，举办公益演出100场、广场文化活动200场，放映公益数字电影22000场以上。

　　整合文化、广电、新闻出版资源，基本完成市属艺术院团、非时政类报刊出版单位改革。

　　奥林匹克公园完成主体工程。创建3个国家级健身中心。高标准完成“十二冬会”长春赛区承办任务并力争取得优异成绩。

　　推进社会主义核心价值体系建设，大力弘扬“宽容大气、自强不息”的城市精神，努力营造知荣辱、讲正气、作奉献、促和谐的良好风尚。

　　把荣获“全国文明城市”称号作为新的起点，更加深入持久地开展精神文明创建活动，推动城市文明程度不断实现新的飞跃。

　　（六）全力保障和改善民生

　　明年，我们要顺应人民群众过上更好生活的新期待，高标准制定实施第六个民生行动计划，再办一批让人民群众看得见、得实惠的好事实事。

　　实施更加积极的就业政策，开发就业岗位13.5万个，实现下岗失业人员再就业4万人，城镇登记失业率控制在4%以内。健全职工工资正常增长、集体协商和支付保障机制，努力增加职工收入。继续提高个体工商业户、小型微型企业税费起征点，强化创业培训，增加创业贷款，推动创业成为富民的重要途径。

　　加强价格监测和市场调控，建立群众生活必需品动态储备机制，保持物价总水平基本稳定。低保补助标准与物价水平挂钩联动，切实保障困难群众基本生活。

　　城镇职工、城镇居民、被征地农民、农村居民等各类养老保险新增参保人数200万以上，尽快实现城乡养老保险全覆盖。进一步提高退休人员养老金发放标准，确保养老保险按时足额发放。改制企业退休人员社会化托管服务范围扩大到20万人，力争再用一年时间实现应管尽管。

　　提高城区集中供养“三无”人员生活补贴和农村五保户供养标准。增加民办养老机构运营补贴。为社区老年日间照料站提供运营补贴。全面落实老年人各项优惠政策。长春已进入老龄化社会，我们必须抓紧建立养老服务社会化体系，努力让老年人生活得更有保障、更加幸福。

　　大幅度提高职工医保、居民医保、新农合报销限额。职工医保、居民医保新增20个定额治疗病种，新农合实施医保“议价谈判”，努力降低群众医疗费用支出。四县（市）和双阳区中心医院实施基本药物制度。推行乡（镇）卫生院托管村卫生所制度，进一步提高基层医疗服务能力。

　　城区贫困家庭脑瘫、听障6岁以下儿童医疗救治实现“有一助一”。 进一步降低贫困残疾人医保起付线，提高医保报销比例。为贫困残疾人家庭子女提供扶残助学金。免费为残疾人提供职业技能培训。优先为贫困残疾人家庭安排保障性住房。新建残疾人康复中心和一批社区康复服务站，加强城市无障碍设施建设，努力为残疾人平等参与社会生活创造条件。

　　新建续建1万套廉租房、1.7万套公租房，4000套廉租房、6000套公租房年内交付使用。拆除160万平方米棚户区和危旧房。无籍房确权500万平方米。高标准、高质量地完成1000万平方米老旧楼宇“暖房子”改造。粉刷5500栋住宅楼道。综合整治一批“老旧散”小区。全面普查高层住宅弃管电梯，建立维修保养长效机制。

　　大力发展学前教育，新建改建38所公办幼儿园，支持民办幼儿园健康发展。推进大学区建设，均衡义务教育资源，全面实施素质教育，进一步提高教育教学质量。继续实施特殊教育三年发展规划，努力让残疾孩子享受一流的特殊教育。全面落实《校车安全条例》，多渠道、多形式解决中小学校车问题，让校车成为安全的“流动校舍”。启动建设体育职业技术学院、合隆职业教育园区，做大做强职业教育。建立和完善贫困家庭子女从学前教育到高等教育全程资助的助学政策，决不让这些孩子因贫失学。

　　实施健康行动计划，创建国家健康城市。深入开展高危致病因素干预、重点疾病早期筛查，积极防治慢性病。扶持促进中医药事业发展。新建扩建儿童医疗中心、市中心医院、市中医院、市心理医院。新建急救指挥平台、4个急救站，7个急救站进驻消防站，努力提高城市急救能力。

　　尽快把流动人口就业、医保、社保、子女就学、优生筛查等方面服务纳入基本公共服务体系。改革户籍管理制度，允许城区具备稳定居所、稳定职业的流动人口申请落户。及时救助生活无着落流浪乞讨人员。

　　（七）加强和创新社会管理

　　进一步简政放权，强化区级政府社会管理职能。积极推动街道办事处和社区工作转型，把主要精力转到社会管理和改善民生上来，努力把社会矛盾化解在基层和萌芽状态。加强社会组织的服务和管理，充分发挥社会力量在社会管理中的积极作用。

　　市民信访接待大厅建成启用，政府职能部门进驻实施联合接访，为信访群众提供一站式服务。进一步完善局长接待日制度，每个月市政府都要指定一个工作日，由政府各部门、县（市）区、开发区以及市政公用单位主要负责人面对面地接待群众，掌握民情、化解矛盾、促进和谐。切实办好市长公开电话，积极回应新闻媒体监督，努力为群众办实事、解难题。

　　深刻吸取朝阳区湖西街道长久家苑违法强制拆迁案件的教训，依法从严打击违法强制拆迁行为，建立长效机制，切实维护被拆迁群众合法权益。

　　积极开展“六五”普法，进一步提高全民法治观念。引导群众以理性合法的形式，表达利益诉求，解决利益纠纷，促进社会和谐。

　　深入推进科技强警，全面落实社会治安打、防、控措施，严厉打击各类刑事犯罪行为，坚决做到黄赌毒必治、命案必破、黑恶势力必除，绝不允许任何一种治安问题成风，绝不允许黑恶势力滋生做大。

　　全面提升食品药品安全问题的事前防范能力，尽快形成覆盖生产、加工、流通、消费环节的全过程监管体系。对食品药品安全违法行为“零容忍”，出重拳，用重典，坚决依法惩处。

　　继续开展安全建设年活动，深入排查整改各类安全隐患，从源头遏制重特大事故发生。进一步健全应急物资储备和救援队伍，全面提高城市防灾减灾能力。

　　进一步做好民族宗教工作。深入开展双拥共建，巩固军政军民团结。

　　各位代表，明年是本届政府任期的最后一年。我们不仅要全面完成届初确定的各项目标，更要为长春未来发展打下好基础。我们肩上的责任重大，必须比以往任何时候都要重视和加强政府自身建设。我们一定努力做到：

　　更加自觉地接受市人大及其常委会的法律监督和工作监督，主动接受市政协民主监督，认真听取民主党派、工商联、无党派人士和各人民团体的意见。

　　更加积极地推进依法行政，全面推行政务公开，不断深化行政审批制度改革，严格行政执法责任追究，努力提高软环境质量。

　　更加深入地开展党风廉政建设，从源头上预防腐败，严肃查处违法违纪案件，坚决纠正损害群众利益的不正之风，努力建设干净干事的公务员队伍。

　　更加勤勉地开展工作，不畏难、不松劲、不懈怠，始终保持积极进取、昂扬向上的精神状态，以更多的付出报答长春人民对我们的信任和重托。

　　各位代表，新的一年马上就要开始了，我们即将踏上充满希望的新征程。让我们在市委的坚强领导下，万众一心，开拓奋进，共同创造长春更加美好的明天！
